# Transforming Empathy Through Simulation: A Mixed-Methods Study on Dementia Education

**DOI:** 10.1093/geroni/igaf122.1666

**Published:** 2025-12-31

**Authors:** Suzanne Parkman, Julie Larouche, Ashely Condon, Abou El-Makarim Aboueissa

**Affiliations:** University of Southern Maine, Portland, Maine, United States; University of Southern Maine, Portland, Maine, United States; University of Southern Maine, Portland, Maine, United States; University of Southern Maine, Portland, Maine, United States

## Abstract

Dementia, including Alzheimer’s disease, poses substantial challenges due to its progressive and multifaceted nature. Addressing these complexities requires healthcare students to undergo specialized training emphasizing empathy and equipping them with the skills necessary to provide compassionate, patient-centered care. This study used an embedded mixed-methods, one-group quasi-experimental design to evaluate empathy levels in pre-licensure nursing and graduate occupational therapy students (N = 125) using the Kiersma-Chen Empathy Scale-Revised (KCES-R). Empathy data was gathered at three intervals: before, immediately after, and six weeks following a virtual dementia simulation. Additionally, 36 focus groups (comprising 3-4 students each) were conducted during post-simulation debriefings to collect qualitative insights. The findings demonstrated significant increases in empathy scores across all time points, F (2,124) = 17.02, p = 0.000. Thematic analysis of the qualitative data revealed five core themes: 1) The Illusion of Empathy, 2) Building Empathetic Competence, 3) Facing Difficult Realities, 4) The Eureka Moment: Transformative Insights, and 5) Empathy within Power Dynamics. Collectively, the results indicate that simulation-based dementia education effectively fosters empathy among healthcare students while inspiring them to deliver higher-quality care. Experiential learning approaches like these are vital for preparing the next generation of healthcare professionals to meet the growing needs of individuals living with dementia.

